# Strength and sustainability of recycled alkali-activated concrete across temperature and curing regimes

**DOI:** 10.1038/s41598-025-18371-6

**Published:** 2025-09-12

**Authors:** Gehad Mokhtar, Mohammad M. El Basha, Shimaa Younis

**Affiliations:** 1 Civil Engineering Department, Future High Institute of Engineering in Fayoum, Cairo, Egypt; 2 Structural Department, Faculty of Engineering, Ain Shams University, Cairo, Egypt

**Keywords:** Geo-polymer concrete, Recycled aggregate, Sustainable materials, Mechanical properties, Microstructure, Curing temperature, Alkaline activators, Environmental sciences, Engineering, Materials science

## Abstract

The construction industry increasingly adopts sustainable materials and strategies to mitigate its environmental impact. This study evaluates geopolymer concrete incorporating recycled aggregates (GOCRA) as an eco-friendly alternative to conventional cement-based materials. A cement-free geopolymer binder composed of aluminosilicate-rich waste materials and alkaline activators (Na₂SiO₃/NaOH) was used to prepare mixtures containing natural coarse aggregate (basalt) and recycled coarse aggregates (RCA), including crushed concrete (CC) and crushed ceramic (CE). Specimens were cured at 40 °C, 60 °C, and 80 °C for 60 h to assess the influence of curing temperature on compressive strength development. The results showed that geopolymer concrete incorporating recycled aggregates exhibited comparable mechanical performance to control mixes with natural aggregates. The highest compressive strength was recorded when curing at 60 °C for 60 h, reaching 41.5 MPa in the reference mix and approximately 37–38 MPa in mixes containing crushed concrete and crushed ceramic. These results confirm that recycled aggregates can produce sustainable geopolymer concrete with good strength. Scanning electron microscopy revealed a dense microstructure with strong interfacial bonding. These findings highlight the potential of geopolymer concrete with recycled aggregates to achieve both environmental benefits and satisfactory mechanical properties. A novel approach is presented for estimating the porosity of crushed aggregate mixtures using ImageJ software, providing a simple and cost-effective alternative to traditional laboratory methods.

## Introduction

Concrete is the most widely used construction material globally due to its strength, durability, and versatility in various structural applications. It plays a central role in infrastructure development, including buildings, transportation networks, and hydraulic structures^1,2^. However, the conventional cement industry is among the largest contributors to environmental pollution, primarily due to the substantial CO₂ emissions generated during production and calcination processes^3–5^. Moreover, the accumulation of ceramic waste from manufacturing plants and the massive quantities of demolished concrete debris, particularly in regions affected by armed conflicts, have created significant challenges for waste management and environmental sustainability. These issues intensify resource depletion and contaminate soil, water, and air^6,7^. Geopolymer concrete has emerged as a sustainable alternative to conventional cementitious materials due to its ability to utilize industrial by-products such as fly ash, ground granulated blast furnace slag, and calcined clays as aluminosilicate binding agents, significantly reducing carbon emissions compared to ordinary Portland cement^8–11^. Another critical parameter affecting the performance of geopolymer concrete is the concentration of the alkaline activator solution, which governs the geopolymerization reaction and impacts both fresh and hardened properties^12,13^. In addition to the environmental benefits of alkali-activated binders, replacing natural aggregates with recycled materials such as crushed concrete and ceramic waste promotes resource conservation. It supports effective waste management strategies^14–18^. Recent studies have highlighted that alkali-activated concrete can achieve excellent mechanical performance, demonstrating high compressive strength and favorable microstructural characteristics. These properties are primarily attributed to the development of dense reaction products and strong bonding within the matrix, which enhance the material’s structural integrity and durability^19–21^. Moreover, various studies have explored the influence of different industrial by-products and recycled materials on the mechanical behavior of alkali-activated binders. Incorporating fly ash, slag, biomass ash, and recycled asphalt waste has enhanced compressive and tensile strength and durability under freeze–thaw cycles and chloride penetration. Such advances contribute to developing more sustainable and high-performance concrete composites^22–25^. Curing temperature and duration are critical parameters that strongly influence the development of mechanical properties and microstructure in alkali-activated concretes. Elevated curing temperatures have been reported to accelerate geo-polymerization reactions, enhance early-age strength, and improve matrix densification^26,27^. However, excessively high temperatures or prolonged curing can lead to increased porosity, shrinkage cracking, and long-term durability concerns^28^. Additionally, the durability of concrete under extreme temperature conditions has been comprehensively investigated through mechanical and fracture mechanics approaches^29,30^. Consequently, optimizing curing regimes is essential to achieving balanced performance and ensuring consistent quality in sustainable concrete mixtures^31^. Although many studies have investigated alkali-activated systems with recycled aggregates, limited research has systematically addressed the combined influence of curing regimes, temperature variations, and recycled aggregate incorporation on mechanical and microstructural development^32^. This study aims to fill this gap by evaluating recycled alkali-activated concrete’s strength, durability, and sustainability subjected to different curing temperatures and durations. The outcomes are expected to provide valuable insights into mix design strategies that optimize environmental benefits and mechanical performance in recycled alkali-activated concrete.

## Materials and methods

### Materials

**Fig. 1:**
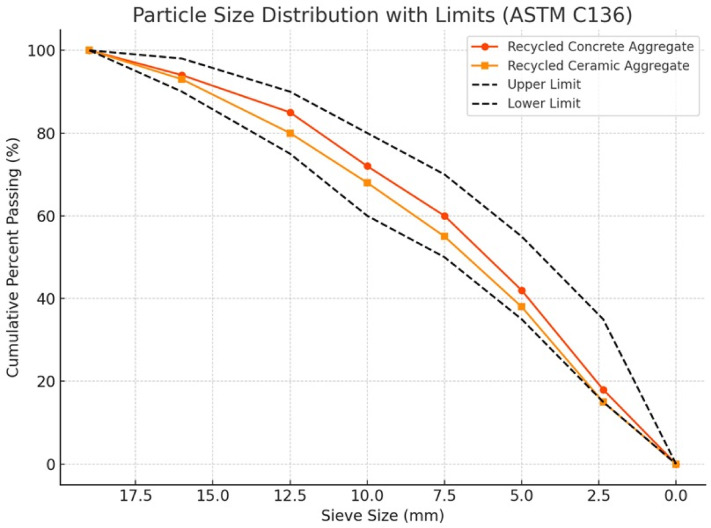
Shows the results of the Sieve analysis for recycled coarse aggregates.

Basalt is a naturally occurring coarse aggregate with a maximum size of 19 mm, a Specific Gravity of 2.64, and an absorption of 0.8%. Recycled ceramic aggregate is manufactured from crushed ceramic tiles and sanitaryware waste, whereas recycled concrete aggregate is made by physically crushing waste concrete parts from demolished structural components. The visible pollutants manually removed from both types included dust, gypsum particles, and organic materials. To ensure consistent moisture levels, the components were oven-dried at 105 °C for 24 h before use. ASTM C127 was used in the experimental assessment of water absorption values^33^. Because of its lower porosity, recycled ceramic aggregate absorbed 12.1% more than recycled concrete aggregate, which absorbed 5.7% on average. The particle size distribution investigation was performed using a set of standard sieves by ASTM C136^34^, with sieve openings ranging from 19 mm to 0.150 mm as shown in Fig. 1. An image-based method for evaluating changes in particle size and morphology distributions of aggregate materials after a vibratory compaction test. Crushed concrete with a max size of 19 mm, a specific gravity of 2.5, crushed ceramic with a max size of 19 mm, and a specific gravity of 2.12 were made from recycled coarse aggregates. Fine aggregate (sand) with a specific gravity of 2.55 and a fineness modulus of 2.55. We measured the aggregate crushing value (ACV) according to British Standard 812. Higher crushing resistance was shown by recycled ceramic aggregate, which ranged from 20 to 26%, and recycled concrete aggregate, which had ACV values ranging from 25 to 32%^35^. found comparable results.

Fresh tap water. Sika ViscoCrete_3425 has a density of 1.08 kg/lit and 2% of the weight of slag. The optimized blended Geopolymer concrete mix made with a binder content (slag) of 450 kg/m^3^, alkaline activator was created by a combination of sodium hydroxide (NaOH) 30% and sodium silicate (Na₂SiO₃) 70%. The activator from the sodium silicate solution (Na_2_O = 12%, SiO_2_ = 30%, and water = 57% by mass) and sodium hydroxide (NaOH) in flakes or pellets form with 99% purity, the concentration of the sodium hydroxide solution used is 12 molars (M) without additional water. Solution to binder ratio of 0.38.

### Mix design

Various GOCRA mix designs were developed, incorporating different proportions of recycled aggregate. The binder-to-aggregate ratio and alkaline solution concentrations were optimized to ensure good workability and strength. Several replacement levels (0% and 100%) of natural aggregates with recycled aggregates were considered, and three concrete mixtures were produced to cast samples. Table 1 shows mixture proportions.

**Table 1:** Design of the mixtures (kg/m^3^).

Mix No.	Slag	Sand	Aggregate	sodium hydroxide	sodium silicate	Water reducer
Go-T	450	623.66	1247.31	51.3	119.7	9
Go-CC	450	601.45	1202.92	51.3	119.7	9
Go-CE	450	536.89	1043.77	51.3	119.7	9

Go-T-Refer to traditional geo-polymer concrete mixture with coarse aggregate (basalt).

Go-CC-Refer to geo-polymer concrete mixture with recycled coarse aggregate (crushed concrete).

Go-CE-Refer to geo-polymer concrete mixture with recycled coarse aggregate (crushed ceramic).

### Testing procedures

According to ECP 203, compressive strength was tested on 150 × 150 × 150 mm cubes at 7, 28, 56, and 90 days. The compressive strength after 28 days was measured at various curing durations and temperatures (40℃, 60℃, 80℃). Tensile strength was measured using cylinders that were 150 mm in diameter and 300 mm in height. A static flexural test was conducted 28 days after the specimens were cast. This test followed the ASTM C78 standard for simple beams subjected to third-point loading. The beams were 100 × 100 × 500 mm. The load was applied to the specimen at the third point using an apparatus that fit the specifications of the ASTM standard. For each variable, a minimum of three specimens were created. Microstructural analysis was performed using Scanning Electron Microscopy (SEM) to study the microstructure of the geopolymer binder and its interaction with recycled aggregate. Small fragments (~ 10 mm) were taken from the fractured surface of the tested cubes/beams, dried, and gold-coated before examination.

## Results and discussion

### Compressive strength

**Fig. 2:**
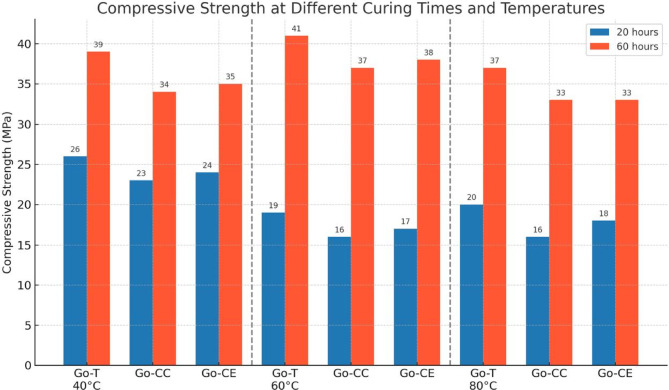
Compressive strength of Alkali-Activated Concrete at different temperatures with curing time. Go-T: Refer to traditional geo-polymer concrete mixture with coarse aggregate (basalt). Go-CC: Refer to geo-polymer concrete mixture with recycled coarse aggregate (crushed concrete). Go-CE: Refer to geo-polymer concrete mixture with recycled coarse aggregate (crushed ceramic).

The values for the 28th day of the different temperatures (40, 60, and 80℃) and curing times were calculated for each combination using three concrete cubes. As shown in Fig. 2, compressive strength data show that samples cured at 60 °C for 60 h had the best strength. Control geo-concrete specimens had the highest increase in compressive strength (41.5 MPa), followed by geo-recycled concrete (CE) at 38.1 MPa and geo-recycled concrete (CC) at 37.3 MPa. While heat curing at 60 °C increases early strength, extended curing at higher temperatures (e.g., 90 °C) can decrease mechanical strength because of increasing porosity and micro-defects in the gel phase, according to a study on high-strength metakaolin-based geopolymer composites^36,37^. A 60-hour curing period allows enough time for structural densification and gel formation to create a strong geopolymer matrix. According to research, heat curing speeds up strength development in the early stages. Still, prolonged exposure to high temperatures can cause the precursor gel products to shrink and split, which disrupts the matrix and prevents the development of strength later on^37^. Curing at 60 °C produces a denser microstructure with fewer pores and fissures than lower or unreasonably high temperatures. The mechanical interlocking between particles is improved by this densification, increasing the load-bearing capability. According to studies, raising the curing temperature increases the geopolymer matrix’s density and continuity while decreasing air spaces and microcracks, increasing strength^38^.

Curing geopolymer concrete for 60 h at 60 °C achieves an ideal balance. This speeds up the polymerization process for an early strength gain while preventing the microstructural flaws that come with higher temperatures or longer curing times. In light of this, the behavior of strengths was investigated at 7, 56, and 90 days after specimens were cured at 60 °C for 60 h. The compressive strength of geopolymer concrete specimens is presented in Table 2.

The compressive strength results differ between Go-T, Go-CC, and Go-CE. The recycled aggregate mixes (Go-CC and Go-CE) exhibited delayed strength growth but outstanding long-term performance, whereas Go-T had the highest early-age strength (41.5 MPa at 28 days). At 28 days, Go-CC (37.3 MPa) and Go-CE (38.1 MPa) showed lower strength than Go-T. But after 56 days, Go-CE outperformed Go-T in terms of 28-day strength (43.8 MPa vs. 41.5 MPa), showing that recycled materials can perform on par with or better with longer curing times. Continuous strength gains for up to 90 days. Go-CC and G0-CE recycled aggregates (42.12 MPa and 44.32 MPa, respectively). The compressive strength increase follows the results achieved by^38,39^ because of gel Maturation and continued geo-polymerization. Unreacted aluminosilicate components still dissolve and contribute to creating new geo-polymeric gel phases even after initial curing^40^. The extended geo-polymerization process in geopolymer concrete specimens increased their compressive strength by 5–9% between 56 and 90 days. According to the investigation^41^, the ongoing growth of the polymeric structure in the geopolymer matrix is responsible for the 2–5% strength rise between 28 and 56 days. Including certain materials in geopolymer concrete aids moisture retention, promoting prolonged strength development^42^. Over extended periods, the alkali content within geopolymer concrete stabilizes, reducing the risk of alkali-related degradation mechanisms. This stabilization contributes to the material’s durability and strength retention^43^.

On the other hand, Go-CC (37.3 MPa) and Go-CE (38.1 MPa) showed somewhat lower early strength because of the recycled aggregates’ higher porosity, which first absorbs alkaline activators and delays geo-polymerization^44^, and the residual mortar on the recycled aggregates, which results in a weaker ITZ and lowers early load-bearing capacity^45^. The performance of recycled aggregates can be comparable with longer drying durations. Go-CE (43.8 MPa) exceeded Go-T’s 28-day strength by 56 days, while Go-CC (41.77 MPa) nearly matched it. This is in line with studies that show slower geo-polymerization in recycled mixes results in a delayed but ongoing strength gain^46^. The remaining aluminosilicate phases in recycled aggregates react over time to promote densification, which improves recycled aggregates for up to 90 days. Meanwhile, pozzolanic reactions from recycled fines may densify microcracks, enhancing long-term durability^47^. The results indicate that using ceramic waste as recycled aggregate in geopolymer concrete achieves higher compressive strength than recycled concrete aggregate. This is attributed to the stable mineralogical composition of ceramic material and its rough surface, which enhances bonding with the geopolymer matrix and its chemical stability in alkaline environments. These properties lead to a denser matrix and stronger internal bonds, thereby improving mechanical performance. These findings are consistent with recent literature, such as the study by^48^.

**Table 2:** Compressive strength results at different ages (MPa).

Mix No.	7 days	28 days	56 days	90 days
Go-T	30.5	41.5	48.14	48.91
Go-CC	26.5	37.3	41.77	42.12
Go-CE	25.8	38.1	43.8	44.32

Go-T: Refer to traditional geo-polymer concrete mixture with coarse aggregate (basalt)

Go-CC: Refer to geo-polymer concrete mixture with recycled coarse aggregate (crushed concrete).

Go-CE: Refer to geo-polymer concrete mixture with recycled coarse aggregate (crushed ceramic).

### Tensile and flexural strength

**Fig. 3:**
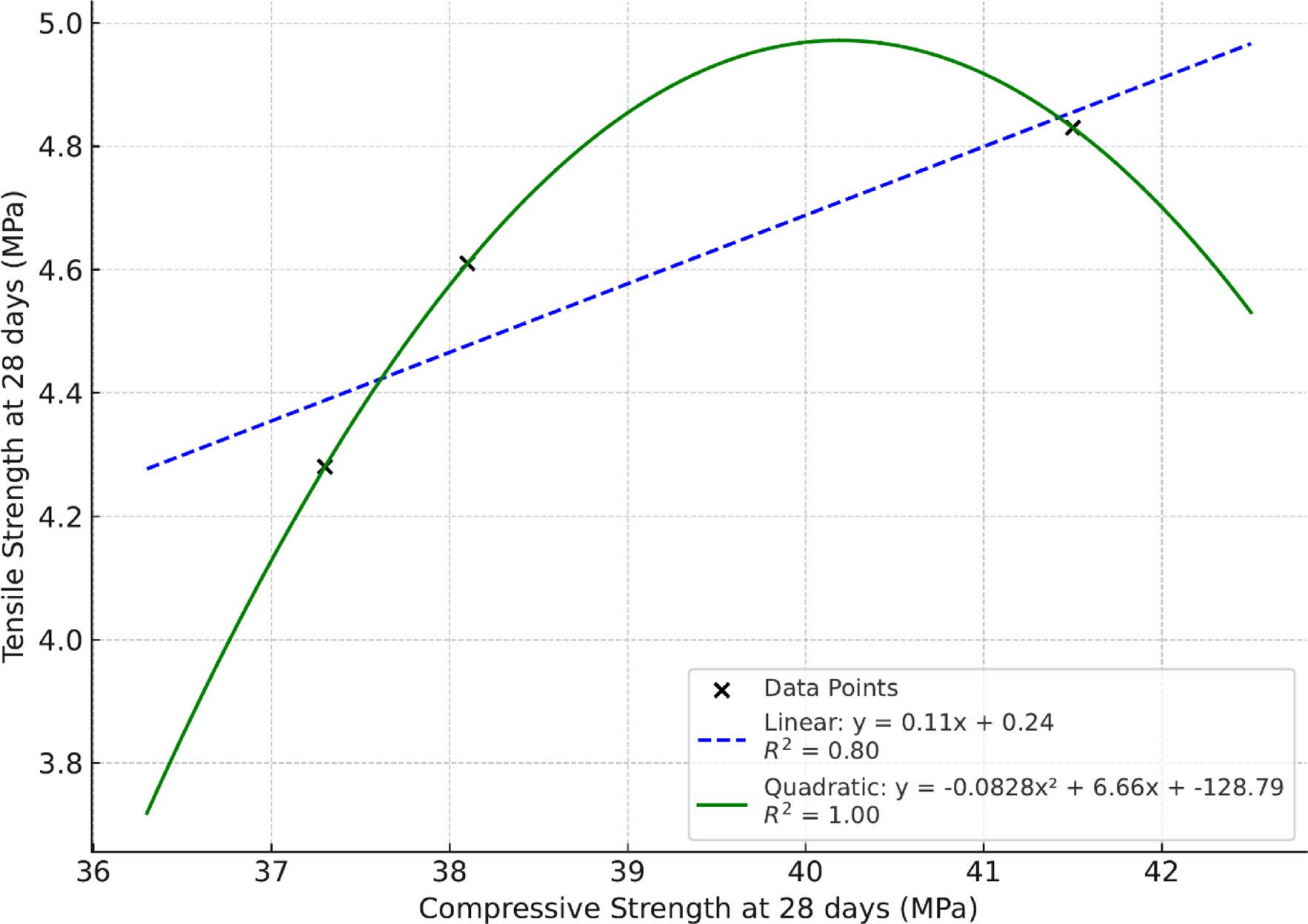
Comparison between compressive and tensile strengths at 28 days.

**Fig. 4:**
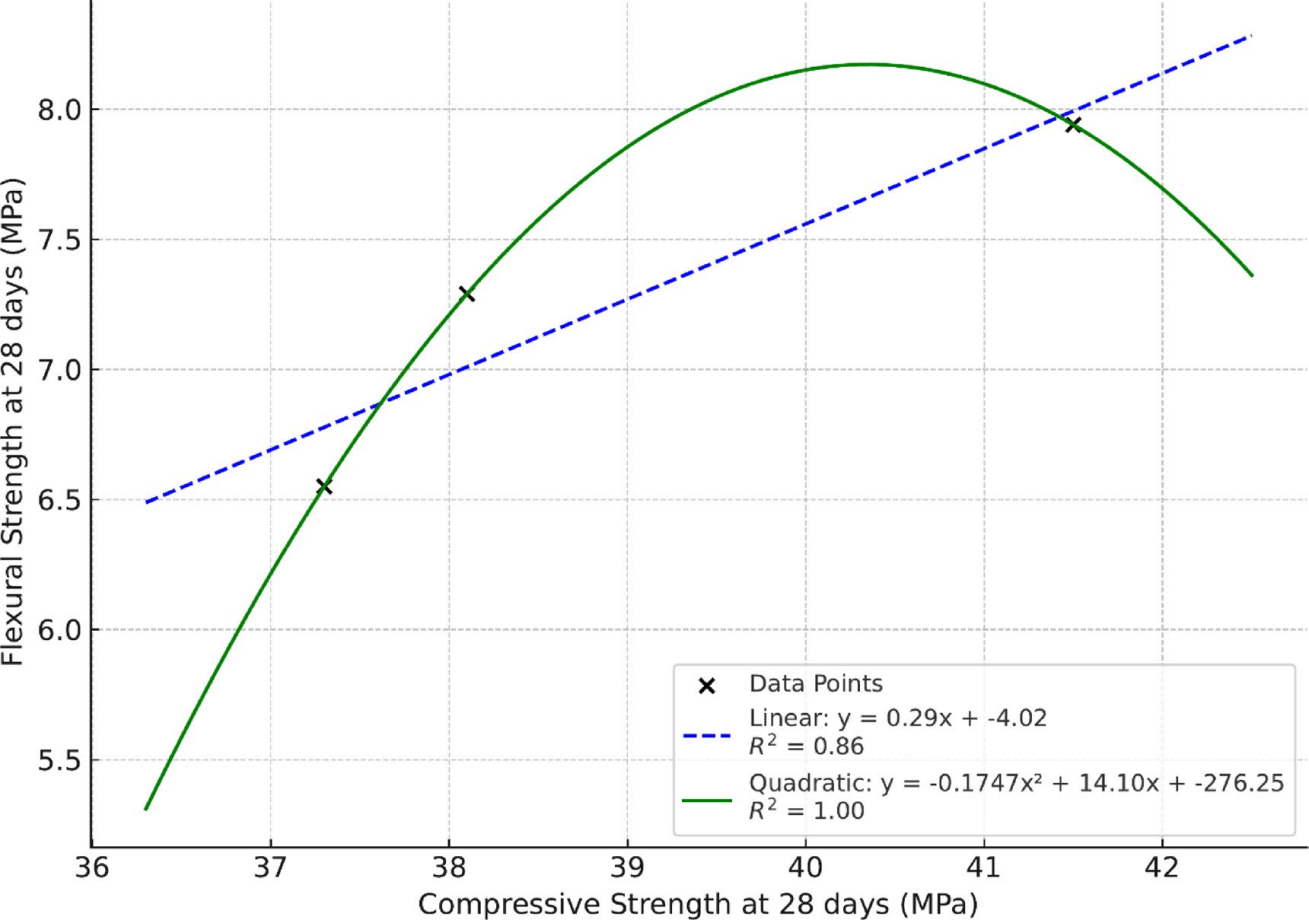
Comparison between compressive and flexural strengths at 28 days.

Split tensile and flexural strengths of geopolymer concrete specimens are presented in Table 3. The strength improvement follows^49^ reports that GOC has superior ITZ bonding, contributing to better load transfer and tensile behavior. A strongly cross-linked three-dimensional aluminosilicate network (Si–O–Al links) is created via geo-polymerization, improving crack resistance, stress distribution, and gradually raising tensile and flexural strength^50^. Low porosity achieved in GOC leads to better mechanical performance, including flexural strength^51^. Based on these findings and the improvement in the mechanical properties of concrete, a comparison was conducted between compressive strength and tensile strength, as shown in Fig. 3 as well as compressive strength and flexural strength, as shown in Fig. 4.

**Table 3:** Tensile and flexural strength results at 28 days (MPa).

Mix No.	Tensile strength	Flexural strength	
Go-T	4.83	7.94	
Go-CC	4.28	6.55	
Go-CE	4.61	7.29	

Go-T: Refer to traditional geo-polymer concrete mixture with coarse aggregate (basalt).

Go-CC: Refer to geo-polymer concrete mixture with recycled coarse aggregate (crushed concrete).

Go-CE: Refer to geo-polymer concrete mixture with recycled coarse aggregate (crushed ceramic).

### Microstructural analysis

**Fig. 5:**
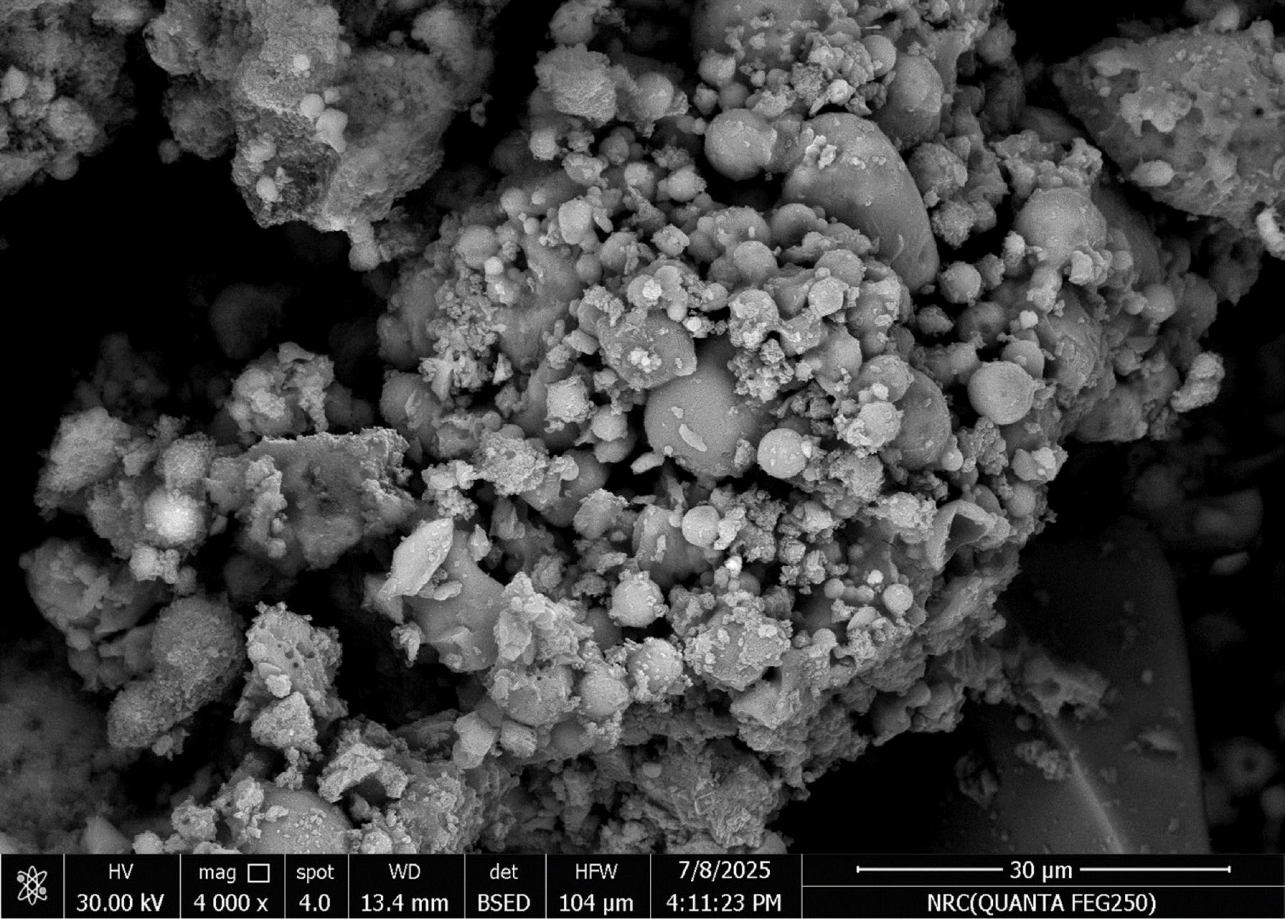
SEM micro-image shows high porosity and low strength of the interfacial interaction between the matrix and recycled crushed aggregates.

**Fig. 6:**
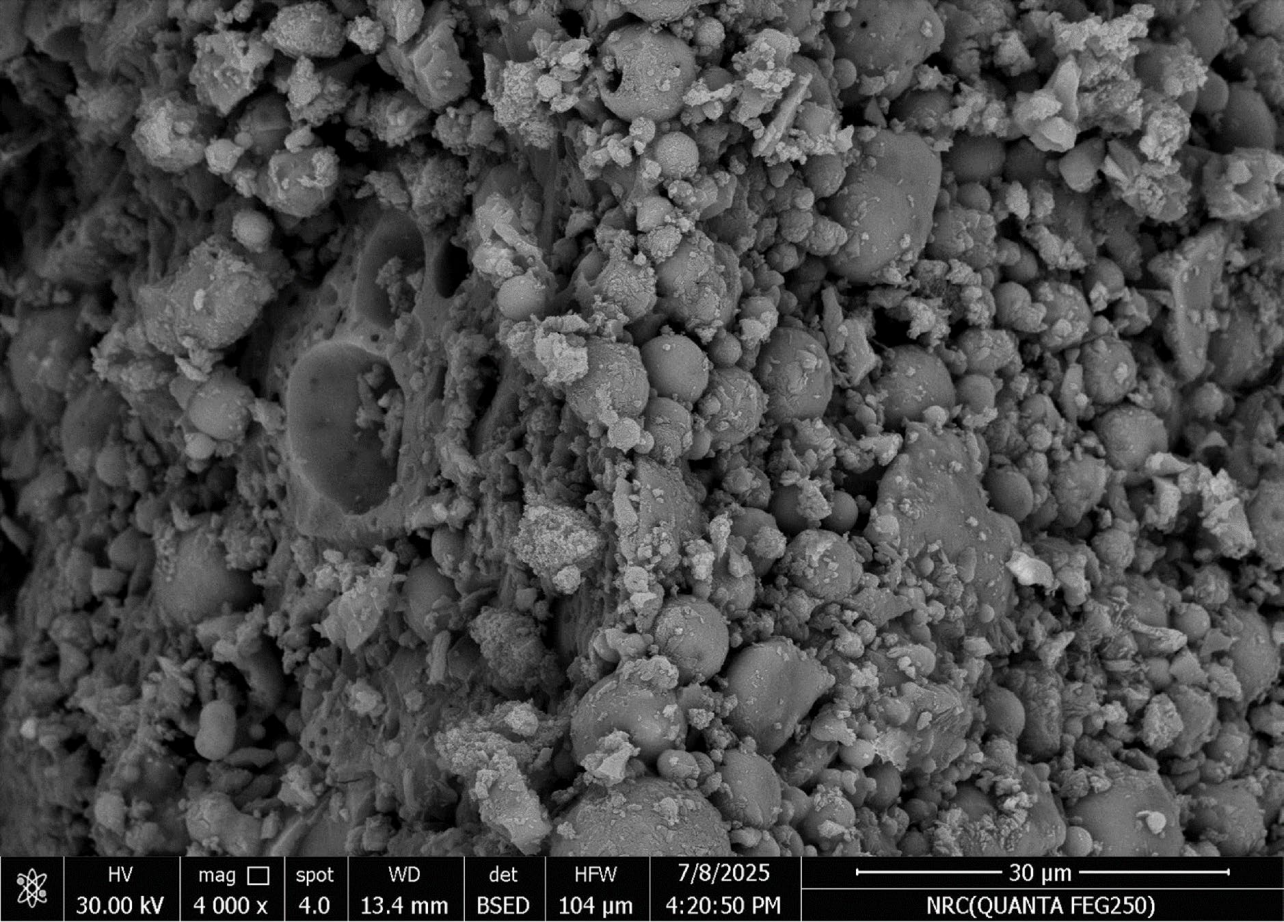
SEM micro-image shows low porosity and high strength, strengthening the interfacial interaction between the matrix and recycled ceramic aggregates.

The microstructure of geopolymer concrete gets increasingly compact over time as gel phases form and rearrange themselves. As geo-polymerization progresses, aluminosilicate gels such as N-A-S-H (sodium aluminosilicate hydrate) and C-A-S-H (calcium aluminosilicate hydrate) form and reorganize, filling voids within the matrix. This results in a denser, more compact microstructure. GOCRA specimens were screened and quantified using EDX-SEM analysis, as shown in Figs. 5 and 6. Using Image J software, the porosity of the samples was determined by looking at the binarized SEM pictures. The photos were converted to 8-bit grayscale and thresholded to distinguish pores from the solid matrix. The area fraction of pores was used to calculate total porosity^52^. Recycled concrete aggregate (Go-CC) in geopolymer concrete had a total porosity of 18.0%, according to the porosity measurements. In contrast, recycled ceramic aggregate (Go-CE) in the mix had a lower porosity of 15.1%. The microstructural characteristics seen in the SEM pictures of the two blends agree with these results. The Go-CC had a more porous and heterogeneous microstructure, with visible weak interfacial transition zones (ITZ), unreacted particles, and microcracks. This structure can be due to old cement paste adhering to recycled crushed particles, which frequently produces micro-defects and compromises the continuity of the geopolymer matrix. In comparison, the Go-CE mixture had a more compact and homogenous microstructure, as shown in the SEM image. The rough and reactive surface of the recycled ceramic particles most likely facilitated better bonding with the geopolymer gel, resulting in improved ITZ quality and reduced pore connectivity. The lower measured porosity of 15.1% reflects this densification and indicates better long-term durability^53^.

.

## Conclusion

This study indicates the viability of using geopolymer concrete with recycled ceramic aggregates as a sustainable and technically sound alternative to conventional and recycled concrete aggregate combinations. The ceramic-based combination demonstrated improved mechanical behavior and better microstructural characteristics, indicating its potential for structural applications. Furthermore, the valorization of ceramic waste helps to reduce environmental effects and promotes circular economy practices in the building sector. The findings also highlight the need to use recycled aggregates in alkali-activated systems to reduce reliance on natural resources. This strategy is consistent with existing attempts to produce low-carbon construction materials that fulfill technical performance standards while addressing environmental sustainability issues.

## Data Availability

No datasets were generated or analysed during the current study.
